# A neuromechanics-based powered ankle exoskeleton to assist walking post-stroke: a feasibility study

**DOI:** 10.1186/s12984-015-0015-7

**Published:** 2015-02-25

**Authors:** Kota Z Takahashi, Michael D Lewek, Gregory S Sawicki

**Affiliations:** Joint Department of Biomedical Engineering, North Carolina State University and University of North Carolina at Chapel Hill, 911 Oval Drive, Campus Box 7115, Raleigh, NC 27695 USA; Division of Physical Therapy, Department of Allied Health Sciences, University of North Carolina at Chapel Hill, Bondurant Hall, 321 South Columbia St, Campus Box 7135, Chapel Hill, NC 27599 USA

**Keywords:** Stroke, Exoskeleton, Gait, Rehabilitation, Ankle

## Abstract

**Background:**

In persons post-stroke, diminished ankle joint function can contribute to inadequate gait propulsion. To target paretic ankle impairments, we developed a neuromechanics-based powered ankle exoskeleton. Specifically, this exoskeleton supplies plantarflexion assistance that is proportional to the user’s paretic soleus electromyography (EMG) amplitude only during a phase of gait when the stance limb is subjected to an anteriorly directed ground reaction force (GRF). The purpose of this feasibility study was to examine the short-term effects of the powered ankle exoskeleton on the mechanics and energetics of gait.

**Methods:**

Five subjects with stroke walked with a powered ankle exoskeleton on the paretic limb for three 5 minute sessions. We analyzed the peak paretic ankle plantarflexion moment, paretic ankle positive work, symmetry of GRF propulsion impulse, and net metabolic power.

**Results:**

The exoskeleton increased the paretic plantarflexion moment by 16% during the powered walking trials relative to unassisted walking condition (p < .05). Despite this enhanced paretic ankle moment, there was no significant increase in paretic ankle positive work, or changes in any other mechanical variables with the powered assistance. The exoskeleton assistance appeared to reduce the net metabolic power gradually with each 5 minute repetition, though no statistical significance was found. In three of the subjects, the paretic soleus activation during the propulsion phase of stance was reduced during the powered assistance compared to unassisted walking (35% reduction in the integrated EMG amplitude during the third powered session).

**Conclusions:**

This feasibility study demonstrated that the exoskeleton can enhance paretic ankle moment. Future studies with greater sample size and prolonged sessions are warranted to evaluate the effects of the powered ankle exoskeleton on overall gait outcomes in persons post-stroke.

**Electronic supplementary material:**

The online version of this article (doi:10.1186/s12984-015-0015-7) contains supplementary material, which is available to authorized users.

## Background

For individuals post-stroke, their capacity to walk is often compromised. These individuals, compared to healthy adults, typically walk with slower self-selected speeds [1], greater inter-limb asymmetry [2,3] and elevated metabolic cost [4,5]. While these gait limitations are largely due to the abnormalities in the paretic limb, a notable contributing factor may be the impaired functions of the ankle musculature. The paretic ankle mechanics show impaired joint moment and power generation [6-10]. The ankle joint, in healthy individuals, generate more mechanical energy than any other muscle groups [11] and play a critical role in forward propulsion and swing phase initiation [12]. The diminished ankle ‘push-off’ in individuals post-stroke may therefore contribute to the decreased walking speeds [8,13] and inadequate swing phase mechanics [7,10]. Furthermore, impaired ankle mechanics may lead to a series of compensations elsewhere, including greater reliance on the non-paretic limb [8,14]. An important goal for rehabilitation, then, may be to enhance paretic ankle function to maximize locomotor recovery.

Contemporary post-stroke rehabilitation approaches may include body weight support training to offload a portion of the body’s weight [15,16], split-belt treadmill training [17,18] and sensory feedback presented in a virtual environment [19-21] to improve symmetry and/or increase walking speeds. Other approaches may involve assistive robots designed to aid movement of the lower limb joints (e.g., knee, hip) [22-27]. While these ‘global’ interventions are aimed to assist the whole-body or several lower limb joints, more local interventions have also improved gait outcomes by targeting the ankle impairments. Functional electrical stimulation, for example, has been applied to the paretic ankle plantarflexors in attempt to restore propulsion mechanics [28-30]. Such application can increase propulsive ground reaction forces, increase swing phase knee flexion [29], increase self-selected walking speed and decrease metabolic cost of transport [30]. Similarly, interventions via elastic ankle orthoses can contribute to increased self-selected walking speed [31] and decreased metabolic cost [32,33].

In parallel with existing ‘ankle-centric’ rehabilitation, our goal here was to implement a powered ankle exoskeleton to enhance paretic limb mechanics. While this type of device has been applied previously in persons post-stroke [34], our focus here was to extend this technology such that the exoskeleton interacts directly with the user’s volitional control. An electromyography (EMG) controlled exoskeleton, for example, could provide externally-powered plantarflexion in magnitude proportional to the user’s soleus activity [35,36]. Due to its user-controlled interface, this powered exoskeleton may be an enticing approach to enhance paretic ankle mechanics for post-stroke rehabilitation.

Prior investigations of EMG controlled ankle exoskeletons in healthy individuals have revealed valuable insights onto how users interact with the device [35-38]. One study showed that such exoskeleton can increase total ankle joint power [38], and thus could be viable for post-stroke rehabilitation. Though, the users of EMG controlled exoskeletons also tend to preserve an invariant ankle moment by reducing their soleus muscle activation [38]. This reduced muscle activity may be counterproductive when the inherent goal of post-stroke rehabilitation is to *enhance* ankle mechanics. But for those with already weakened ankle muscles due to stroke, it is unclear how the mechanical assistance will influence user interaction. Among a multitude of possible adaptations, we envision one of three possibilities. First, the users could suppress plantarflexor muscle activity to preserve an invariant total ankle moment (i.e., similar to healthy individuals, albeit with reduced ankle moment). Second, the users could preserve the same muscle activation, and the exoskeleton assistance would enhance the total ankle moment. Lastly, the users could enhance muscle activity during assistance, and thus amplify the total ankle moment. The specific adaptations that the users choose could inform the viability of powered exoskeleton for post-stroke rehabilitation. Therefore, overall goal of this feasibility study was to evaluate the immediate effects of a neuromechanics-driven powered exoskeleton on post-stroke gait. While long-term assessment and training were beyond the scope of this study, we envision that this feasibility study will guide future work in exoskeleton-assisted rehabilitation.

But before implementing EMG-driven exoskeleton for post-stroke application, there is one additional factor to consider. The performance of powered exoskeletons, at least in healthy individuals, is notably sensitive to the timing of mechanical actuation [39]. However, the timing of muscle activation in persons post-stroke could be affected due to factors like spasticity, weakness, and altered coordination [40,41]; and thus these abnormal muscle conditions could complicate the application of purely EMG-controlled exoskeleton. To this end, we have developed a powered ankle exoskeleton that integrates both EMG and ground reaction forces to assist paretic ankle function. Specifically, this exoskeleton provides mechanical assistance proportional to the plantarflexor EMG activity *only* during a phase of gait when the stance limb is subjected to an anteriorly directed ground reaction force. In other words, this exoskeleton retains the myoelectric controller developed previously [35-37], but in addition, controls the timing of actuation based on the onset of propulsion.

With this integrated control algorithm to target propulsion in the paretic limb, we expected to see improved gait outcomes in persons post-stroke. In particular, we hypothesized that the powered ankle exoskeleton would increase paretic ankle plantarflexion moment and power output compared to unassisted walking. Due to the ankle’s role in supplying whole-body propulsion [12] and its purported importance in facilitating physiologically efficient walking [42], we also hypothesized that the enhanced paretic ankle mechanics would improve propulsion symmetry and reduce the metabolic cost of walking.

## Methods

### Proportional Myoelectric Propulsion (PMP) powered exoskeleton

**Figure 1:**
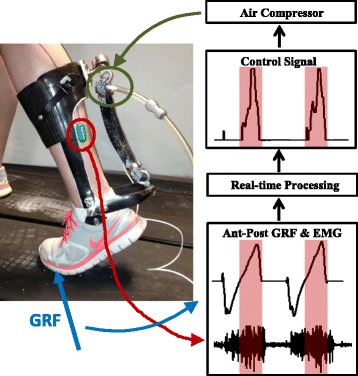
**Illustration of the proportional myoelectric propulsion (PMP) powered exoskeleton.** The soleus electromyography (EMG) and anterior-posterior ground reaction force (GRF) from an instrumented treadmill were collected in real-time to control the magnitude and timing of exoskeleton actuation. The proportional myoelectric propulsion (PMP) controller supplies plantarflexion moment proportional to the soleus EMG activity *only* during a phase of gait when the stance limb is subjected to an anteriorly directed ground reaction force. The red highlighted region denotes the duration in which the exoskeleton is activated.

**Figure 2:**
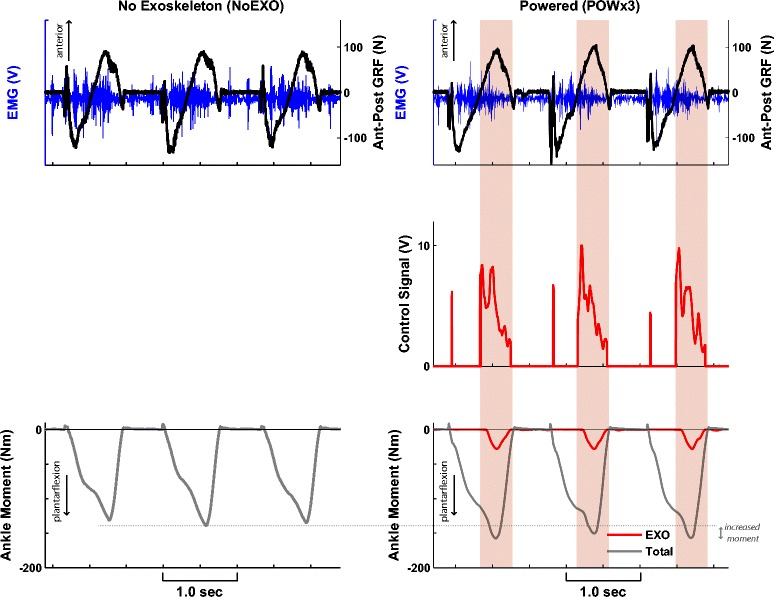
**Paretic limb data (three steps) from a representative subject with and without the powered exoskeleton.** During the powered walking (POWx3), the exoskeleton control signal was generated with magnitude proportional to the paretic soleus EMG (blue) only when the anterior-posterior GRF (black) was greater than 0 (region highlighted in red). Positive GRF denotes anterior (i.e., propulsive) force. During this phase, the exoskeleton supplied plantarflexion moment during late stance (red), contributing to the increased total ankle moment (gray) relative to the NoEXO condition. We note that there is a delay between the onset of the control signal and the onset of the exoskeleton moment (lag of approximately 83 ms).

We fabricated a lightweight ankle exoskeleton for each individual’s paretic limb (Figure 1). The exoskeleton consisted of a custom-fitted carbon fiber shank and foot components hinged at an ankle joint (total device mass = 532.3 ± 72.0 g). An artificial pneumatic muscle (length = 26.0 ± 6.0 cm) was attached along the posterior shank (moment arm = 13.4 ± 1.5 cm) to provide a plantarflexion moment about the ankle. The magnitude and timing of the exoskeleton assistance was based on the subjects’ paretic soleus EMG signal (Biometrics, Newport, UK) and ground reaction force (GRF) data from an instrumented treadmill (Bertec, OH, USA), collected and processed in real-time. For the real-time processing (data sampled at 960 Hz), the raw EMG signal was high-pass filtered with a 2^nd^ order dual-pass Butterworth filter (50 Hz cutoff frequency), full-wave rectified, and low-pass filtered with a 2^nd^ order dual-pass Butterworth filter (10 Hz cutoff frequency), while no filtering was applied to the GRF data. We implemented a proportional myoelectric propulsion (PMP) control algorithm, in which the exoskeleton supplied plantarflexion moment proportional to the paretic soleus EMG signal *only* during the phase of stance when the anterior-posterior GRF was greater than 0 (Figure 1). In essence, the PMP controller attempts to enhance the functions of the ankle plantarflexors during the propulsive phase of gait, under the user’s volitional action (Figure 2 and Additional file 1: Movie).

### Experimental protocol

We recruited 5 subjects with stroke (subject characteristics are listed in Table 1). Each subject walked on an instrumented treadmill in the following order: (1) walking without an exoskeleton (i.e., NoEXO), (2) walking with an exoskeleton without powered assistance (i.e., UnPOW), and (3) walking with powered exoskeleton assistance (i.e., POW). The exoskeleton was worn only on the paretic limb. Each condition lasted for 5 minutes (with 5 minutes of rest in between), and each subjected repeated the POW condition three times for a total of 15 minutes. We set the treadmill speed to approximately 75% of the subjects’ preferred speed. This preferred speed was determined by instructing the subjects to ‘walk at your comfortable speed’ over a straight walkway for 10 meters (averaged across three repetitions). We set the gain and threshold of the proportional myoelectric component of the PMP controller during the UnPOW condition such that the control signal saturated for at least three consecutive steps. Once the gain and threshold were determined, these parameters remained constant during the three POW conditions.

**Table 1:** **Subject characteristics**

**Subject**	**Age**	**Height (m)**	**Body mass (kg)**	**Paretic limb (L or R)**	**Months since stroke**	**Preferred speed (m/s)**	**Treadmill speed (m/s)**
S1	57	1.70	99.9	L	357	1.09	0.80
S2	51	1.72	79.3	L	53	0.72	0.50
S3	82	1.78	98.0	R	134	0.57	0.40
*S4	69	1.86	89.6	L	212	0.85	0.60
S5	47	1.91	126.0	R	102	0.98	0.70
**Average**	**61.2**	**1.79**	**98.6**		**171.6**	**0.84**	**0.60**
**Std**	**14.3**	**0.09**	**17.4**		**118.7**	**0.21**	**0.16**

*S4 wore his prescribed ankle-foot orthosis (Allard ToeOFF) during the NoEXO condition.

For all conditions, the subjects wore an overhead safety harness, but the harness itself was not intended to supply body weight support. All subjects were discouraged from using the handrails mounted bilaterally on the treadmill, but we note that two of the subjects were not able to complete the protocol without support from the handrails. During the NoEXO condition, subjects walked with their own shoes. One subject (S4, see Table 1) wore his clinically-prescribed ankle-foot orthosis (Allard ToeOFF) on his paretic limb during the NoEXO condition. No other subjects in this study had a prescribed ankle-foot orthosis.

### Data analysis

We used an eight-camera motion analysis system (Vicon, Oxford, UK) to capture kinematic data (120 Hz), and the instrumented treadmill to collect kinetic data (960 Hz) during walking trials. We used a 6 degree-of-freedom marker set [43] to track lower extremity motion. To analyze the forces generated by the exoskeleton, we mounted a compression load cell (Omegadyne, OH, USA) in-series with the pneumatic muscle (collected at 960 Hz). We applied a 2^nd^ order dual-pass low-pass Butterworth filter (6 Hz for kinematic data, and 25 Hz for kinetic data). For all GRF, joint mechanics, and EMG outcome variables, we analyzed the last minute (at least 10 steps of data) of each walking condition.

All GRF variables were analyzed by normalizing to body weight (BW). We computed the time integral of anterior-posterior GRF to quantify the braking and propulsion impulse (BW*sec). Percent paretic propulsion was quantified as the ratio of the paretic propulsion impulse divided by the sum of the paretic and non-paretic propulsion impulses [44].

Sagittal plane joint angle, moment (Nm kg^−1^), and power (W kg^−1^) at the ankle, knee, and hip were computed using Visual3D software (C-Motion, Germantown, MD). Positive and negative mechanical work (J kg^−1^) performed by the individual joints were computed by integrating the positive and negative portions of the joint power data with respect to time, respectively. During the UnPOW and POW conditions, the contribution of exoskeleton to the total ankle moment was quantified by the product of the forces recorded from the pneumatic muscle and the exoskeleton moment arm. Likewise, the contribution of exoskeleton to the total ankle power was obtained by the product of the exoskeleton moment and the ankle joint angular velocity.

We collected surface EMG data of soleus (SOL) and tibialis anterior (TA) muscles from both paretic and non-paretic limbs (collected at 960 Hz). The EMG signals were high-pass filtered with 2^nd^ order dual-pass Butterworth filter (20 Hz), rectified, and low-pass filtered with a 2^nd^ order dual-pass Butterworth filter (10 Hz) to create a linear enveloped EMG. We then computed the time integral of the processed EMG signal during the propulsion phase of stance. The integrated EMG (iEMG) signal for each muscle across all conditions was normalized to its magnitude during the NoEXO condition.

A portable metabolic system (Oxycon Mobile, Viasys Healthcare, CA) was used to record rates of oxygen consumption and carbon dioxide production during the walking trials. Before the walking trials, a 5 minute quiet standing trial was collected to estimate rate of metabolic energy consumption during standing. For standing and all walking trials, metabolic data from the last 2 minutes were averaged, and rates of oxygen consumption and carbon dioxide production were converted to metabolic power using equations Brockway’s equation [45]. Net metabolic power (W kg^−1^) during the walking trials was estimated by subtracting metabolic power during standing from metabolic power during walking [46].

### Statistics

We performed statistical tests on the four dependent variables that were related to our hypotheses: (1) peak paretic ankle plantarflexion moment, (2) paretic ankle positive work, (3) percent paretic propulsion, and (4) net metabolic power. One-factor (5 levels: NoEXO, UnPOW, POWx1, POWx2, and POWx3) repeated measures ANOVA was used to test for differences across walking conditions for each of the four variables. F-ratios for main effect were considered significant for p < 0.05. If a significant main effect was found, paired t-tests were used to make pairwise comparisons across the different conditions. Due to the exploratory nature of this feasibility study and limited sample size, we opted not to perform any adjustments for multiple comparisons to control for Type I errors. Additionally, due to technical difficulties with two subjects’ EMG data, we only reported the means and standard deviations across each condition from three subjects. For all other gait-related variables, we reported the means and standard deviations across each condition as supplementary data.

## Results

The data for paretic ankle joint moment and work, percent paretic propulsion, and net metabolic power were based on averaged results over 5 subjects, whereas the data from EMG were averaged results over 3 subjects. Data from all other gait-related variables were included as supplementary data (averaged results over 5 subjects) including ground reaction force and spatiotemporal data (Additional file 2: Table S1), time-series of knee joint mechanics (Additional file 3: Figure S1), time-series of hip joint mechanics (Additional file 4: Figure S2), and summary of joint work (Additional file 5: Table S2).

### Paretic ankle joint mechanics

**Figure 3:**
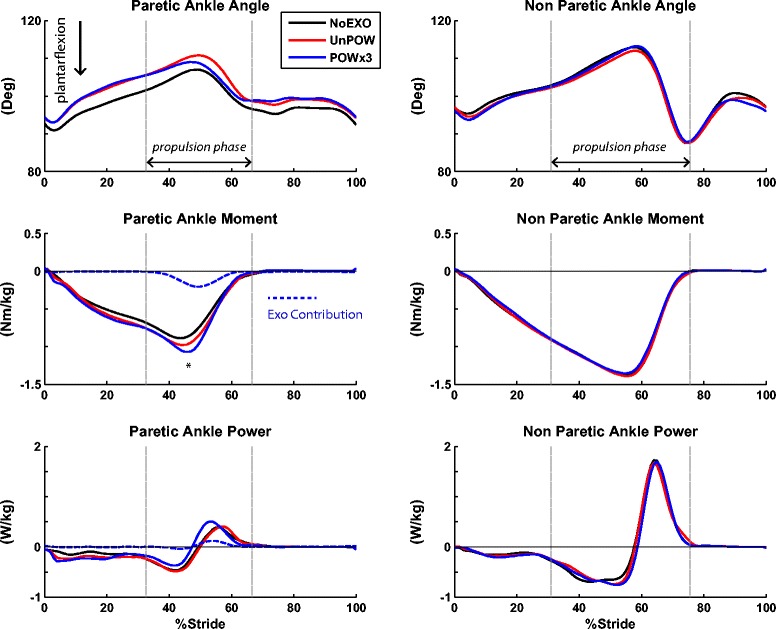
**Ankle joint mechanics (averaged over 5 subjects).** Sagittal plane data (time-normalized to 101 data points across gait cycle) of paretic and non-paretic ankle mechanics (angle, moment, power) were analyzed from the last minute of each condition (NoEXO – black; UnPOW – red; POWx3 – blue). For clarity, data from POWx1 and POWx2 are not shown. The two vertical lines define the propulsion phase of stance (i.e., onset of propulsion and toe-off). During POW conditions, the exoskeleton generated plantarflexion moment during late stance (shown in dotted blue), and contributed to the increased total paretic moment (16% increase during POW relative to NoEXO, p < 0.05).

**Figure 4:**
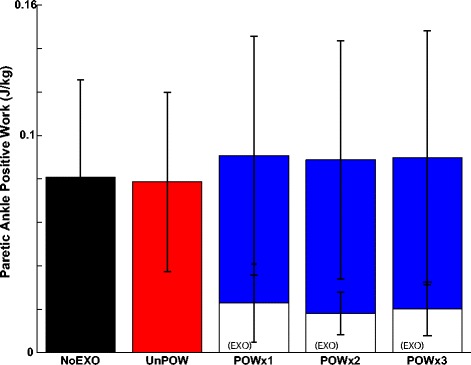
**Paretic ankle positive work (averaged over 5 subjects).** The paretic ankle joint positive work (J kg^−1^) across conditions of NoEXO (black), UnPOW (red), and three repetitions of POW (blue) were analyzed from the last minute of each condition. The exoskeleton’s contributions to the total positive work during POW are denoted in white. There was no statistically significant effect of the exoskeleton on the paretic ankle joint positive work (p =0.58). The error bars represent ± 1.0 standard deviation.

While walking with the exoskeleton (powered or unpowered), the subjects’ paretic ankle was in a more dorsiflexed posture throughout the gait cycle relative to NoEXO (Figure 3). During the POW trials, the exoskeleton remained inactive for the first half of stance, and provided mechanical assistance during late stance. Specifically, the exoskeleton supplied plantarflexion moment of −0.25 ± 0.08 Nm kg^−1^during POWx1, −0.22 ± 0.05 Nm kg^−1^during POWx2, and −0.24 ± 0.05 Nm kg^−1^during POWx3, or approximately 26%, 23%, and 25% of the peak paretic ankle moment during NoEXO, respectively. The exoskeleton initially performed negative work (−0.012 ± 0.018 J kg^−1^ during POWx1, −0.009 ± 0.008 J kg^−1^during POWx2, and −0.007 ± 0.005 J kg^−1^during POWx3), followed by a period of positive work (0.023 ± 0.018 J kg^−1^during POWx1, 0.018 ± 0.010 J kg^−1^during POWx2, and 0.020 ± 0.012 J kg^−1^during POWx3). The exoskeleton had a significant effect on the peak total ankle plantarflexion moment (p = 0.02). The peak ankle moment from all three powered conditions (−1.11 ± 0.32 Nm kg^−1^during POWx1, −1.12 ± 0.29 Nm kg^−1^during POWx2, and −1.11 ± 0.31 Nm kg^−1^during POWx3) were approximately 16% greater that of NoEXO (−0.96 ± 0.32 Nm kg^−1^). Despite the increase in peak paretic ankle moment, there was no significant effect of exoskeleton on ankle positive work (p =0.58) (Figure 4).

### Percent paretic propulsion

**Figure 5:**
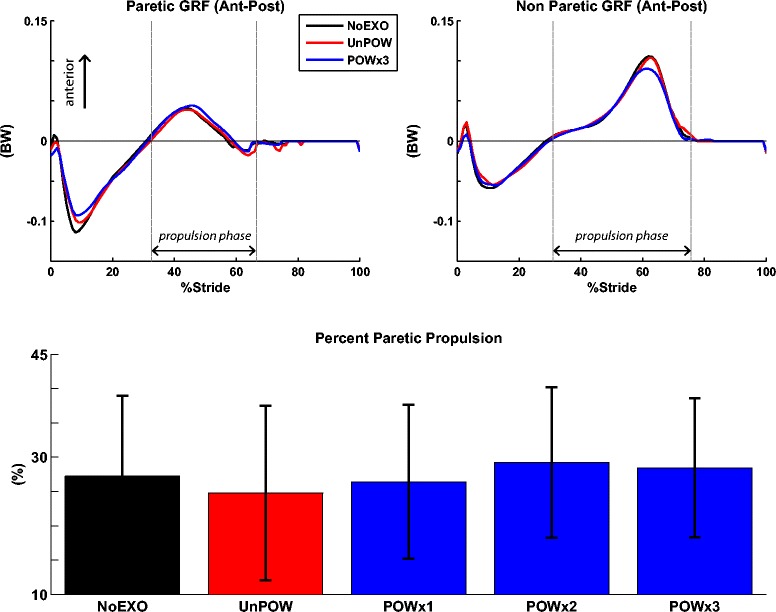
**Anterior-posterior ground reaction force and percent paretic propulsion (averaged over 5 subjects).** Anterior-posterior GRF data (time-normalized to 101 data points across gait cycle) of paretic and non-paretic lower extremities were analyzed from the last minute of each condition (NoEXO – black; UnPOW – red; POWx3 – blue). For clarity, time-series data from POWx1 and POWx2 are not shown. The two vertical lines define the propulsion phase of stance (i.e., onset of propulsion and toe-off). The percent paretic propulsion (described by Bowden et al. [44]) signifies the symmetry of the propulsion impulse (less than 50% indicates greater reliance on the non-paretic limb for propulsion). There was no statistically significant effect of the exoskeleton on the percent paretic propulsion (p = 0.81).

During the NoEXO condition, the percent paretic propulsion was 27.3 ± 11.7%, indicating that the subjects relied more on the non-paretic limb for propulsion (50% indicates perfect symmetry) (Figure 5). The exoskeleton did not affect the percent paretic propulsion, as no significant differences were detected across the conditions (p = 0.81).

### Net metabolic power

**Figure 6:**
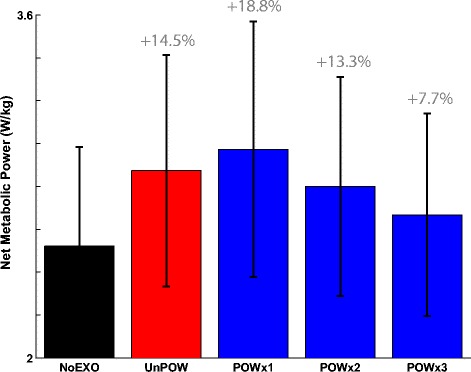
**Whole-body net metabolic power (averaged over 5 subjects).** Whole-body net metabolic power (W kg^-1^) across conditions of NoEXO (black), UnPOW (red), and three repetitions of POW (blue) were analyzed. Although there was no statistically significant effect of the exoskeleton on net metabolic power (p = 0.21), there was a tendency for a gradual reduction of metabolic cost with each bout of the powered walking conditions. The percent change values are expressed relative to the NoEXO condition. The error bars represent ± 1.0 standard deviation.

During the NoEXO condition, subjects expended metabolic energy at a rate of 2.52 ± 0.46 W kg^-1^ (Figure 6). The net metabolic power increased by 14.5% (2.87 ± 0.54 W kg^-1^) during UnPOW. With the addition of the powered assistance, the net metabolic power appeared to successively decrease with each repetition, where the net metabolic power were 2.97 ± 0.60 W kg^-1^ (POWx1), 2.80 ± 0.51 W kg^-1^ in POWx2), and 2.67 ± 0.47 W kg^-1^ (POWx3). However, the differences across all conditions were not statistically significant (p = 0.21).

### Electromyography

**Figure 7:**
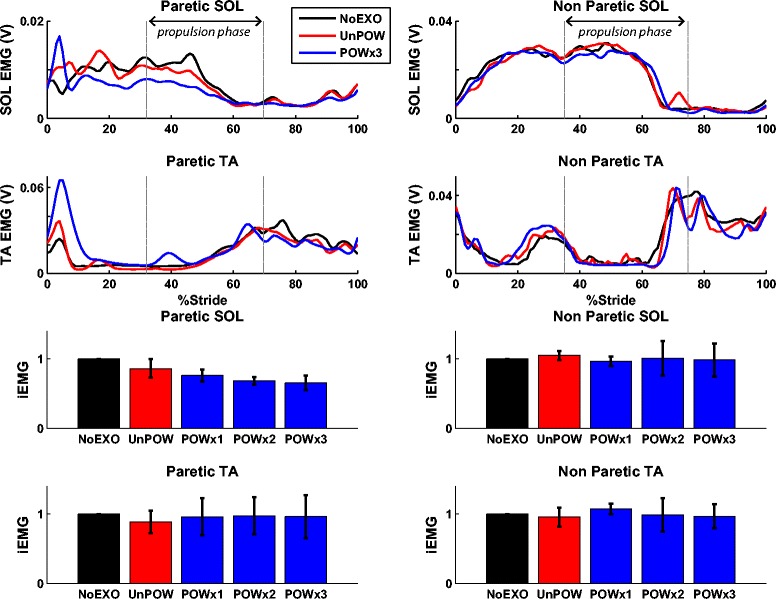
**Linear-enveloped EMG and magnitude of time-integrated EMG during the propulsion phase (averaged over 3 subjects).** EMG signals of SOL and TA (from paretic and non-paretic limbs) were analyzed during the last minute of each condition. Linear-enveloped EMG data (time-normalized to 101 data points across gait cycle) from NoEXO (black), UnPOW (red), and POWx3 (blue) are shown (but linear-enveloped EMG data for POWx1 and POWx2 are not for clarity). The two vertical lines define the propulsion phase of stance (i.e., onset of propulsion and toe-off). The magnitude of integrated EMG (iEMG) during the propulsion phase showed reduced paretic SOL activity (i.e., muscle that controlled the exoskeleton assistance) during all three POW conditions relative to NoEXO. The error bars represent ± 1.0 standard deviation. We note that two subjects’ EMG data were omitted due to technical difficulties, and thus we did not perform statistical analysis on EMG data because of the small sample size.

As only three subjects’ EMG data were analyzed, no statistical analyses were performed for these variables. Within the three subjects, the exoskeleton appeared to affect the paretic SOL muscle activation during the propulsion phase of stance (Figure 7). The magnitude of iEMG during UnPOW decreased by 14% relative to NoEXO. With each POW repetition, the iEMG decreased further (24% lower during POWx1, 31% lower during POWx2, and 35% lower during POWx3). The iEMG from all other muscles (paretic TA, non-paretic SOL and TA) while wearing the exoskeleton did not change by more than 12% relative to NoEXO.

## Discussion

In recent years, ankle-based and/or propulsion-targeted interventions have become a common theme in post-stroke rehabilitation [28-30,32-34]. To our knowledge, this is the first study involving persons post-stroke to apply a neuromechanics (EMG and GRF) driven ankle exoskeleton. To assess its viability as a gait intervention tool, we conducted a feasibility study to examine the short-term effects of the exoskeleton on the mechanics and whole-body energetics of walking. While we emphasize that the results are preliminary with a limited sample size (n = 5), we envision that the findings will inform future work in exoskeleton-assisted rehabilitation.

### Enhanced paretic ankle moment via exoskeleton assistance

With our exoskeleton’s inherent strategy to target the propulsion phase of the paretic limb, we hypothesized that the exoskeleton would enhance paretic ankle mechanics (moment and power) relative to unassisted walking. Indeed, we found that the exoskeleton increased the total paretic ankle plantarflexion moment by 16%. The magnitude of the moment generated by our exoskeleton is similar to previous investigations of powered exoskeletons in healthy individuals [39], as well as studies involving persons post-stroke [34] and persons with incomplete spinal cord injury [47]. Due to the ankle’s role in supplying stance limb propulsion [12] and its purported importance in facilitating physiologically efficient walking [42], we had further hypothesized that the enhanced ankle moment during exoskeleton assistance would lead to improved propulsion symmetry and reduced metabolic cost of walking. Despite the enhanced paretic ankle moment, there were no statistically significant effects of the exoskeleton on any other gait-related outcomes (including paretic ankle positive work, percent paretic propulsion, and net metabolic power). While the lack of statistically significant effects may be largely attributed to the low sample size, other factors contributing to the results may include suboptimal timing of exoskeleton actuation and inadequate adaptation during exoskeleton use.

A previous study involving healthy individuals has suggested that the magnitude of exoskeleton power and its metabolic benefit are sensitive to the timing of actuation [39]. The effect of exoskeleton timing on the ankle joint power generation may be conceptualized in the following way. If the onset of actuation is too early, the pneumatic artificial muscle (which is designed exclusively for generating concentric plantarflexion power) may impede the shank’s forward progression over the foot by triggering premature plantarflexion. If the onset is too late, in contrast, the exoskeleton may have limited time for force production to generate adequate power [39]. It is currently unclear, however, how the exoskeleton controller characteristics (timing and magnitude of assistance) should be individualized for a person with propulsion deficits post-stroke. Factors like increased passive muscle stiffness [48-50], spasticity and increased agonist–antagonist coactivation [40] may all influence the shank’s forward progression during stance, further complicating the application of exoskeleton-assisted interventions. In this study, we initiated the timing of actuation at the onset of propulsive ground reaction forces on the paretic limb, but we recognize that this strategy may not be optimal. In future studies, our goal is to conduct controlled experiments using a versatile exoskeleton test-bed that can readily adapt the magnitude and timing of actuation [51,52] to guide future patient-specific rehabilitation with ankle exoskeletons.

We also reiterate that our protocol only applied 15 minutes of powered walking, and that the subjects may not have received ample accommodation time to reach a steady-state interaction with the exoskeleton. When healthy individuals walk with EMG-controlled exoskeletons, the users adapt and learn to delay their soleus activation such that the exoskeleton produces exclusively positive work [36]. But this adaptation required approximately 30 minutes of familiarization (compared to 15 minutes in our study). Furthermore, the amount of familiarization required for metabolic benefit appears to be influenced by the exoskeleton control algorithms [53]. For example, triggering an actuator at a chosen percentage of stride required approximately 20 minutes of familiarization for metabolic reduction beyond unassisted walking [54], whereas a proportional EMG-based controller required approximately 90 minutes [37]. It is unclear how much familiarization is required for individuals post-stroke using our integrated EMG and GRF controlled exoskeletons. Thus, we feel that a follow-up study with multiple repeated sessions is warranted to better evaluate the long-term effects of our intervention.

### Viability of neuromechanics-based exoskeleton for post-stroke rehabilitation

While intent-based control algorithms have become prominent in the application of exoskeleton and rehabilitation devices [22,55,56] there is a potential drawback of such approach for post-stroke rehabilitation. In healthy individuals, EMG-controlled exoskeleton has been shown to promote reduced plantarflexor activation, possibly as an inherent strategy to preserve a normal ankle moment profile [38] or ankle angle trajectory [36]. Although our exoskeleton was able to increase paretic ankle moment in persons post-stroke, three of the subjects had a tendency to reduce paretic soleus activity during use. This outcome may be counterproductive when the goal of the intervention is to enhance ankle moment and power generation. The reduced muscular activity may also limit the potential benefit of this particular exoskeleton for long-term rehabilitation, as proactive user participation has been identified as a key factor in improving locomotor outcomes following training [57,58]. While predicting long-term outcomes based on our preliminary investigation may be difficult, we believe that a few modifications to our powered exoskeleton intervention are warranted in future studies.

To more effectively engage user interaction with the exoskeleton, one potential approach may be to integrate real-time biofeedback. In this study, we gave no formal instructions to educate the users in how to interact with the exoskeleton, and consequently may have undermined the potential benefits of the device. Thus, future modifications may include adding real-time feedback to increase EMG activity [59,60] or propulsive ground reaction forces [61], or more complex incentive/reward based control schemes [52]. These efforts altogether should promote a more proactive post-stroke rehabilitation to accentuate the viability of exoskeleton interventions.

Another consideration is increasing the demand of walking during the exoskeleton assistance. An important goal for exoskeleton intervention, or any assistive technology for that matter, may be to help patients achieve outcomes that are otherwise difficult under their own strengths. In this study, we constrained the treadmill speed to a fixed percentage of the subjects’ comfortable speed (i.e., the speed at which they can walk *without* assistance). We opted to fix the treadmill speed, since myriad mechanical variables (ground reaction force, joint moment and power) are sensitive to walking speed [11,62-64], thereby enabling a direct assessment of the effects of powered exoskeleton on gait performance. Though in hind sight, constraining the treadmill speed may have limited the effects of the intervention. One plausible explanation for the statistically non-significant changes to the gait outcomes (with exception of paretic ankle joint moment) was that the subjects were already able to walk at the particular speed, and therefore did not need additional assistance from the exoskeleton. Other ankle-based interventions like functional electrical stimulation and elastic ankle orthoses have shown the ability to enable faster walking in persons post-stroke [30,32,33]; and thus we feel that the effects of our propulsion-targeting exoskeleton could be magnified if we allowed the subjects to walk faster during use.

While our neuromechanics-based powered exoskeleton in its current form may not be a viable solution as a portable autonomous walking aid, future work could address this issue. Although interventions like functional electrical stimulation and elastic ankle orthoses already exist [29,30,33,65,66], powered exoskeleton intervention could eventually have several advantages. In functional electrical stimulation, the assistance is bounded by the underlying physiological properties of the muscles it acts upon, whereas powered exoskeletons can adapt the magnitude and timing of assistance with various control algorithms and actuator property. Elastic ankle orthoses can only respond passively to the loads exerted by the user, whereas the neuromechanics-based exoskeletons can offer volitional control. But to realize its potential as a permanent walking aid, our current exoskeletons may require modifications and future investigations.

First, the PMP control algorithm may not be ideal for portability due to the requirements of the ground reaction force. An alternative solution may be using a foot switch to identify the propulsion phase of stance [29]. Another important consideration is that our current exoskeleton only assists in plantarflexion and provides no assistance in dorsiflexion. As foot drop (inability to clear the foot during swing) is a common impairment in persons post-stroke [67], it is unclear whether this particular exoskeleton is suitable for persons with inadequate toe clearance. In one of our subjects, he wore a prescribed ankle-foot orthosis during the NoEXO condition (subject S4). While the powered exoskeleton appeared to increase paretic ankle moment and positive work over his prescribed orthosis, the powered assistance also increased his ankle plantarflexion angle at toe-off (Additional file 6: Figure S3). The effects of such ankle mechanics on toe clearance is unclear, and a recent study has shown that issues related to paretic limb advancement are also affected by the knee and hip joints [68]. Future work should thus evaluate the influence of exoskeleton-assisted plantarflexion on paretic limb advancement.

### Limitations

Limitations of this study are small sample size and limited familiarization time with the powered exoskeleton intervention. The orders of the experimental conditions were not randomized. In addition, two of the subjects could not complete the protocol without the use of handrail support. Though, by analyzing the ground reaction force data, we determined that the average magnitude of handrail support in those two subjects were less than 3% body weight (in the vertical direction) and less than 0.4% body weight (in the anterior-posterior directions) across all experimental conditions. Thus it is unlikely that the handrail support significantly affected the overall outcomes of this study. Furthermore, we custom fabricated an exoskeleton for each individual, and the length of the pneumatic muscle varied depending on the persons’ anatomy (e.g., shank length). The amount of force applied by the pneumatic muscles is largely dependent on its lengths [69], and thus, we could not standardize the magnitude of exoskeleton assistance across all subjects.

## Conclusions

This feasibility study showed that our neuromechanic-based powered exoskeleton enhanced paretic ankle moment relative to unassisted walking. Future studies with greater sample size and prolonged sessions are warranted to better evaluate the effects of the exoskeleton on overall gait outcomes. This study will guide future work in exoskeleton-assisted intervention for establishing its viability for post-stroke rehabilitation.

## Additional files

Additional file 1: Movie. A subject walking with the powered ankle exoskeleton at 0.70 m/s. When the power turned ‘on’, the exoskeleton assisted paretic (right) ankle plantarflexion during the propulsive phase of stance. Additional file 2: Table S1. Summary of ground reaction force (GRF) and spatiotemporal data. Additional file 3: Figure S1. Knee joint mechanics (averaged over 5 subjects). Sagittal plane data (time-normalized to 101 data points across gait cycle) of paretic and non-paretic knee mechanics (angle, moment, power) were analyzed from the last minute of each condition (NoEXO – black; UnPOW – red; POWx3 – blue). For clarity, data from POWx1 and POWx2 are not shown here. The two vertical lines define the propulsion phase of stance (i.e., onset of propulsion and toe-off). Additional file 4: Figure S2. Hip joint mechanics (averaged over 5 subjects). Sagittal plane data (time-normalized to 101 data points across gait cycle) of paretic and non-paretic hip mechanics (angle, moment, power) were analyzed from the last minute of each condition (NoEXO – black; UnPOW – red; POWx3 – blue). For clarity, data from POWx1 and POWx2 are not shown here. The two vertical lines define the propulsion phase of stance (i.e., onset of propulsion and toe-off). Additional file 5: Table S2. Summary of joint mechanics data. Additional file 6: Figure S3. Comparison of powered ankle exoskeleton versus prescribed ankle-foot orthosis from a single subject. While walking with the powered ankle exoskeleton (blue), there was greater paretic ankle range of motion (in dorsiflexion and plantarflexion) compared to his prescribed ankle-foot orthosis (black). In addition, the powered ankle exoskeleton showed 19% greater peak plantarflexion moment, 95% greater peak positive power, and 154% greater positive work compared to his prescribed AFO. The contributions of the exoskeleton to paretic ankle moment and power are also shown (dotted blue).

## Abbreviations

AFO: Ankle foot orthosis
BW: Body weight
EMG: Electromyography
iEMG: Integrated electromyography
GRF: Ground reaction force
PMP: Proportional myoelectric propulsion
NoEXO: Walking without exoskeleton
UnPOW: Walking with exoskeleton unpowered
POW: Walking with exoskeleton powered
