# Supplementary material for: A neuromechanics-based powered ankle exoskeleton to assist walking post-stroke: a feasibility study
Source: J Neuroeng Rehabil. 2015 Feb 25;12:23. doi: 10.1186/s12984-015-0015-7 (PMC4367918; doi:10.1186/s12984-015-0015-7)
Supplement: Additional file 2: Table S1. — Summary of ground reaction force (GRF) and spatiotemporal data. [file 12984_2015_15_MOESM2_ESM.pdf]

Summary of GRF and Spatiotemporal Data

		NoEXO	UnPOW	POWx1	POWx2	POWx3
		Mean +/- Std	Mean +/- Std	Mean +/- Std	Mean +/- Std	Mean +/- Std
Propulsion Impulse:	Non Paretic	0.0283 +/- 0.0049	0.0287 +/- 0.0054	0.0278 +/- 0.0054	0.0272 +/- 0.0030	0.0272 +/- 0.0056
(BW*sec)	Paretic	0.0108 +/- 0.0053	0.0096 +/- 0.0056	0.0101 +/- 0.0052	0.0119 +/- 0.0059	0.0111 +/- 0.0052
Propulsion Peak:	Non Paretic	0.113 +/- 0.037	0.111 +/- 0.038	0.110 +/- 0.037	0.107 +/- 0.035	0.105 +/- 0.030
(BW)	Paretic	0.047 +/- 0.018	0.047 +/- 0.024	0.047 +/- 0.020	0.051 +/- 0.022	0.052 +/- 0.024
Braking Impulse:	Non Paretic	-0.014 +/- 0.005	-0.013 +/- 0.004	-0.013 +/- 0.004	-0.014 +/- 0.005	-0.014 +/- 0.005
(BW*sec)	Paretic	-0.024 +/- 0.006	-0.024 +/- 0.007	-0.024 +/- 0.006	-0.024 +/- 0.006	-0.024 +/- 0.006
Braking Peak:	Non Paretic	-0.078 +/- 0.027	-0.075 +/- 0.023	-0.073 +/- 0.023	-0.074 +/- 0.024	-0.075 +/- 0.024
(BW)	Paretic	-0.118 +/- 0.049	-0.107 +/- 0.043	-0.110 +/- 0.037	-0.109 +/- 0.037	-0.106 +/- 0.035
Vertical Impulse:	Non Paretic	0.775 +/- 0.174	0.785 +/- 0.163	0.784 +/- 0.177	0.800 +/- 0.189	0.801 +/- 0.185
entire stance (BW*sec)	Paretic	0.626 +/- 0.187	0.639 +/- 0.175	0.644 +/- 0.183	0.658 +/- 0.174	0.644 +/- 0.160
Vertical Impulse:	Non Paretic	0.437 +/- 0.119	0.453 +/- 0.162	0.432 +/- 0.125	0.455 +/- 0.149	0.439 +/- 0.139
during propulsion (BW*sec)	Paretic	0.273 +/- 0.072	0.269 +/- 0.073	0.298 +/- 0.090	0.303 +/- 0.085	0.292 +/- 0.078
Vertical Peak (1st):	Non Paretic	0.967 +/- 0.053	0.982 +/- 0.041	0.990 +/- 0.044	0.989 +/- 0.036	0.994 +/- 0.035
(BW)	Paretic	1.010 +/- 0.033	1.017 +/- 0.050	1.012 +/- 0.064	0.999 +/- 0.072	1.005 +/- 0.068
Vertical Peak (2nd):	Non Paretic	0.997 +/- 0.019	1.005 +/- 0.020	0.990 +/- 0.018	0.994 +/- 0.027	1.002 +/- 0.022
(BW)	Paretic	0.938 +/- 0.079	0.952 +/- 0.069	0.977 +/- 0.044	0.963 +/- 0.055	0.961 +/- 0.030
Stance Time:	Non Paretic	1.022 +/- 0.270	1.036 +/- 0.251	1.040 +/- 0.272	1.060 +/- 0.2774	1.061 +/- 0.257
(sec)	Paretic	0.913 +/- 0.343	0.937 +/- 0.320	0.931 +/- 0.336	0.962 +/- 0.3278	0.945 +/- 0.302
Step Length:	Non Paretic	0.352 +/- 0.080	0.367 +/- 0.082	0.370 +/- 0.078	0.390 +/- 0.089	0.385 +/- 0.083
(m)	Paretic	0.454 +/- 0.034	0.451 +/- 0.055	0.449 +/- 0.050	0.450 +/- 0.049	0.447 +/- 0.053
Swing Time:	Non Paretic	0.385 +/- 0.117	0.384 +/- 0.106	0.392 +/- 0.117	0.404 +/- 0.113	0.385 +/- 0.122
(sec)	Paretic	0.492 +/- 0.084	0.491 +/- 0.074	0.499 +/- 0.084	0.498 +/- 0.084	0.503 +/- 0.096
Stride Frequency:	(strides min^{-1})	44.85 +/- 10.07	44.12 +/- 10.09	44.25 +/- 10.92	43.45 +/- 11.37	43.91 +/- 12.04
