# Supplementary figures and images for: A neuromechanics-based powered ankle exoskeleton to assist walking post-stroke: a feasibility study
Source: J Neuroeng Rehabil. 2015 Feb 25;12:23. doi: 10.1186/s12984-015-0015-7 (PMC4367918; doi:10.1186/s12984-015-0015-7)

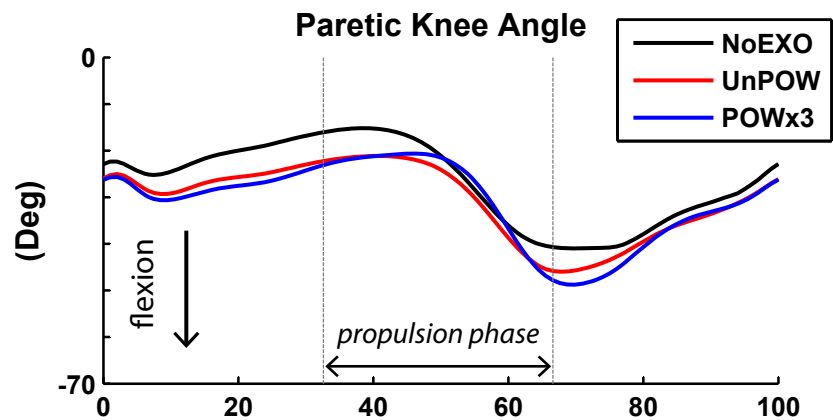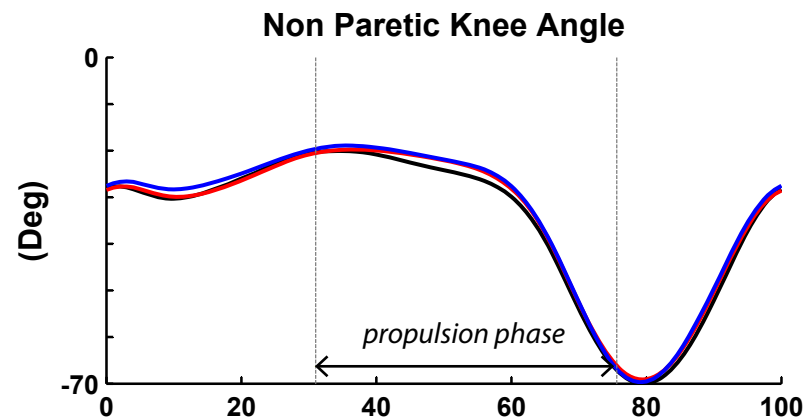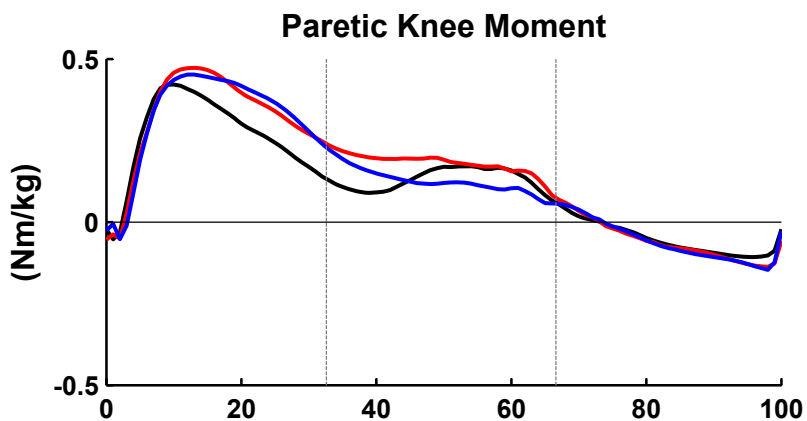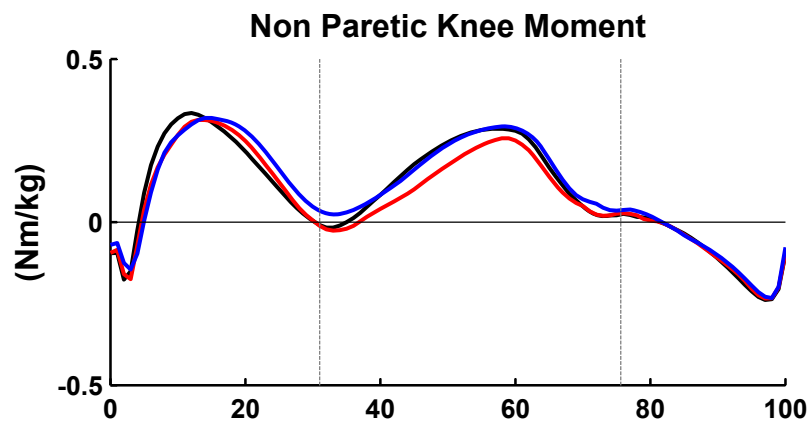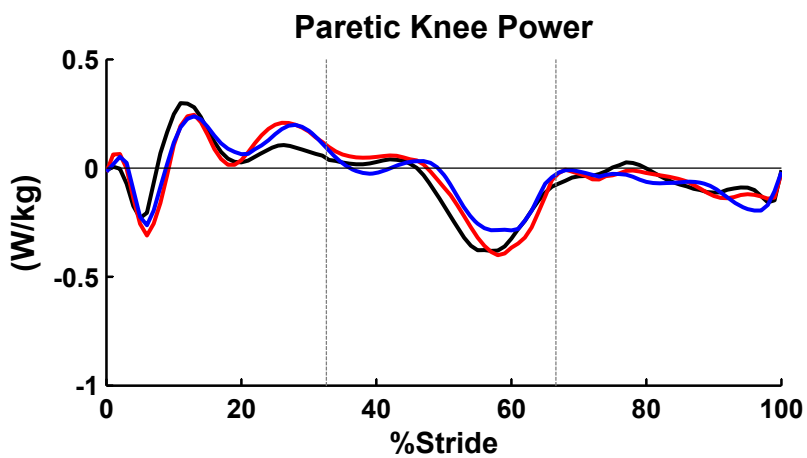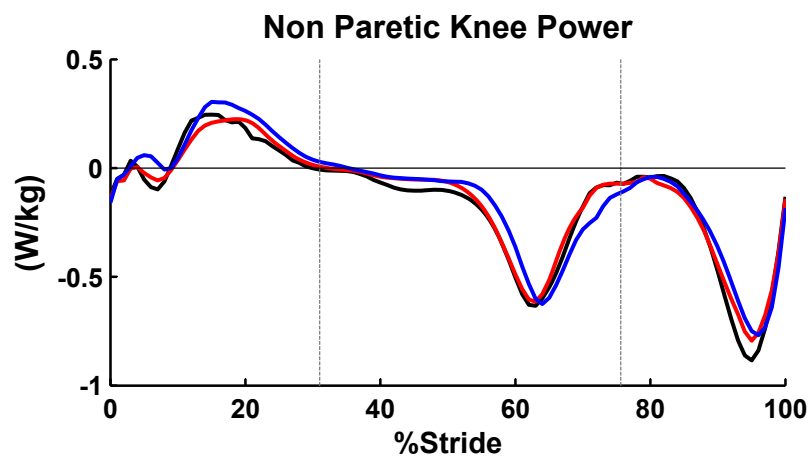

Supplement: Additional file 3: Figure S1. — Knee joint mechanics (averaged over 5 subjects). Sagittal plane data (time-normalized to 101 data points across gait cycle) of paretic and non-paretic knee mechanics (angle, moment, power) were analyzed from the last minute of each condition (NoEXO – black; UnPOW – red; POWx3 – blue). For clarity, data from POWx1 and POWx2 are not shown here. The two vertical lines define the propulsion phase of stance (i.e., onset of propulsion and toe-off). [file 12984_2015_15_MOESM3_ESM.pdf]

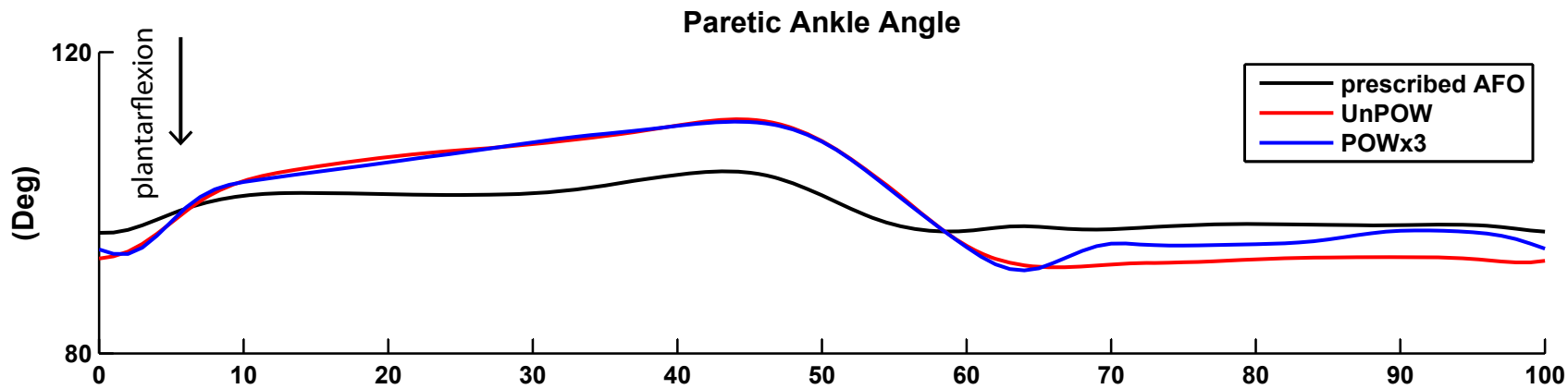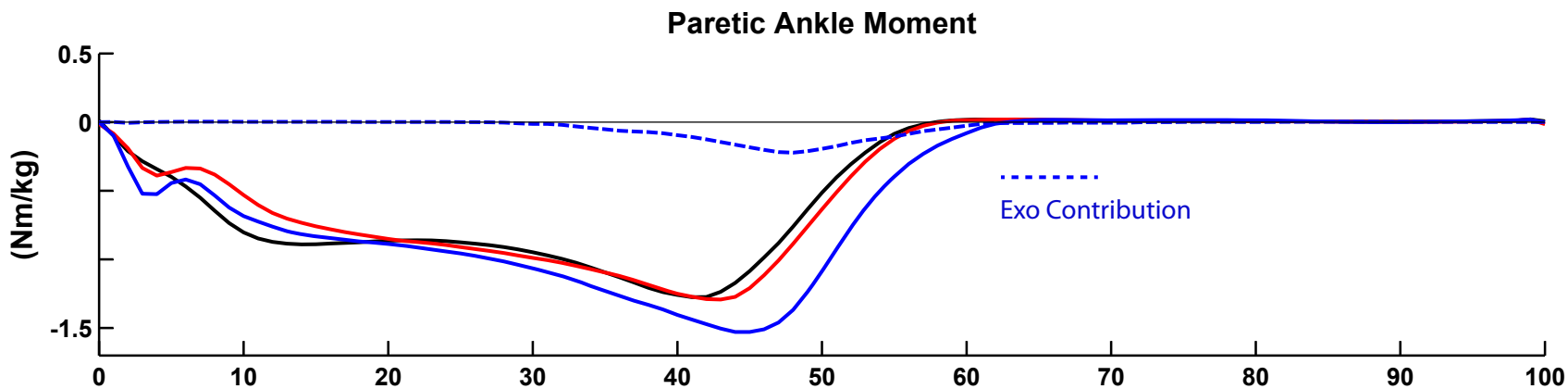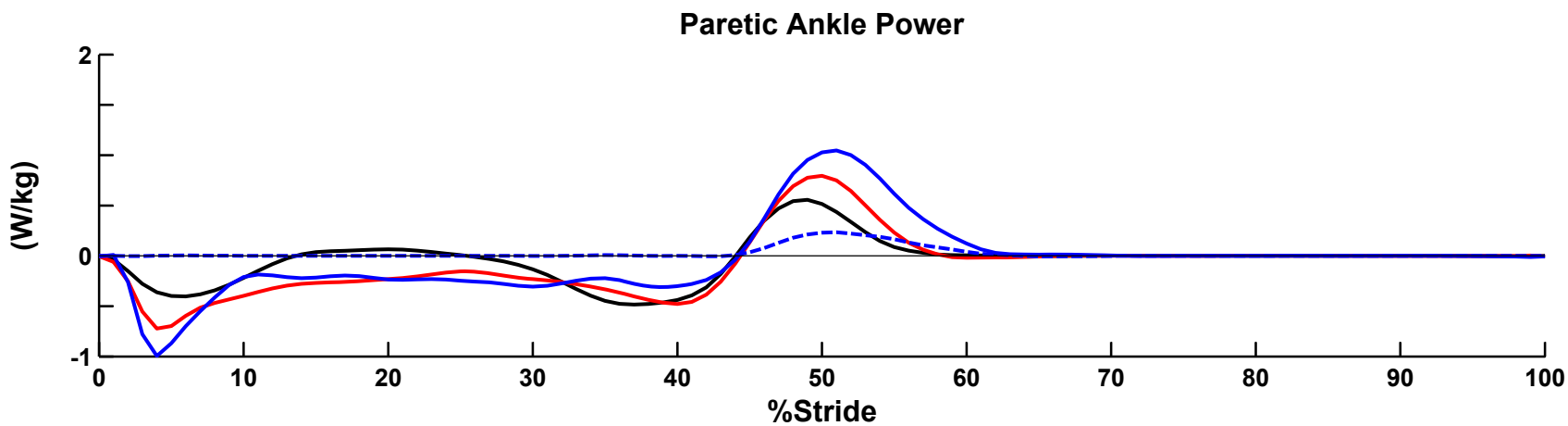

Supplement: Additional file 6: Figure S3. — Comparison of powered ankle exoskeleton versus prescribed ankle-foot orthosis from a single subject. While walking with the powered ankle exoskeleton (blue), there was greater paretic ankle range of motion (in dorsiflexion and plantarflexion) compared to his prescribed ankle-foot orthosis (black). In addition, the powered ankle exoskeleton showed 19% greater peak plantarflexion moment, 95% greater peak positive power, and 154% greater positive work compared to his prescribed AFO. The contributions of the exoskeleton to paretic ankle moment and power are also shown (dotted blue). [file 12984_2015_15_MOESM6_ESM.pdf]
