# Supplementary material for: A neuromechanics-based powered ankle exoskeleton to assist walking post-stroke: a feasibility study
Source: J Neuroeng Rehabil. 2015 Feb 25;12:23. doi: 10.1186/s12984-015-0015-7 (PMC4367918; doi:10.1186/s12984-015-0015-7)
Supplement: Additional file 4: Figure S2. — Hip joint mechanics (averaged over 5 subjects). Sagittal plane data (time-normalized to 101 data points across gait cycle) of paretic and non-paretic hip mechanics (angle, moment, power) were analyzed from the last minute of each condition (NoEXO – black; UnPOW – red; POWx3 – blue). For clarity, data from POWx1 and POWx2 are not shown here. The two vertical lines define the propulsion phase of stance (i.e., onset of propulsion and toe-off). [file 12984_2015_15_MOESM4_ESM.pdf]

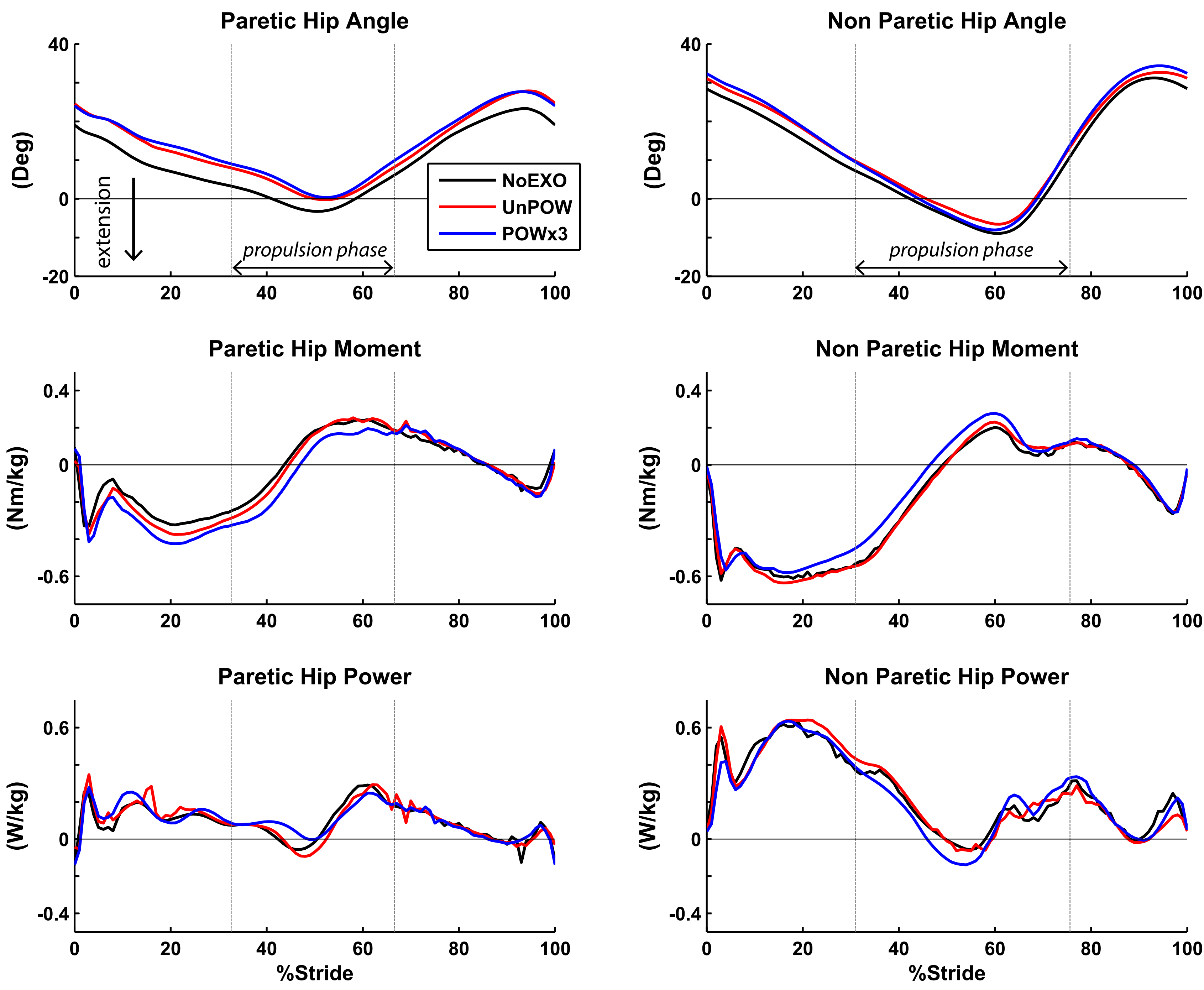

Paretic Hip Angle
Non Paretic Hip Angle
(Deg)
extension
propulsion phase
NoEXO
UnPOW
POWx3
Paretic Hip Moment
Non Paretic Hip Moment
(Nm/kg)
Paretic Hip Power
Non Paretic Hip Power
(W/kg)
%Stride
