# Supplementary material for: A neuromechanics-based powered ankle exoskeleton to assist walking post-stroke: a feasibility study
Source: J Neuroeng Rehabil. 2015 Feb 25;12:23. doi: 10.1186/s12984-015-0015-7 (PMC4367918; doi:10.1186/s12984-015-0015-7)
Supplement: Additional file 5: Table S2. — Summary of joint mechanics data. [file 12984_2015_15_MOESM5_ESM.pdf]

Summary of Joint Mechanics

	NoEXO			UnPOW			POWx1			POWx2			POWx3		
	**Mean**	**+/-**	**Std**	**Mean**	**+/-**	**Std**	**Mean**	**+/-**	**Std**	**Mean**	**+/-**	**Std**	**Mean**	**+/-**	**Std**
**Peak Ankle Moment Non Paretic (Nm kg^{-1})**	-1.405	+/-	0.137	-1.412	+/-	0.169	-1.405	+/-	0.1552	-1.403	+/-	0.1548	-1.401	+/-	0.1781
**Peak Ankle Moment Paretic (Nm kg^{-1})**	-0.957	+/-	0.321	-1.037	+/-	0.252	-1.109	+/-	0.3196	-1.123	+/-	0.2909	-1.111	+/-	0.3145
**Peak EXO Moment (Nm kg^{-1})**	0.000	+/-	0.000	-0.021	+/-	0.015	-0.253	+/-	0.0796	-0.224	+/-	0.0523	-0.240	+/-	0.0490
**Peak Ankle Power Non Paretic (W kg^{-1})**	2.165	+/-	1.190	2.137	+/-	1.157	2.145	+/-	1.1038	2.112	+/-	1.1381	2.029	+/-	1.0080
**Peak Anke Power Paretic (W kg^{-1})**	0.668	+/-	0.277	0.651	+/-	0.222	0.704	+/-	0.4301	0.717	+/-	0.3888	0.730	+/-	0.3817
**Peak EXO Power (W kg^{-1})**	0.000	+/-	0.000	0.012	+/-	0.009	0.189	+/-	0.1519	0.141	+/-	0.0620	0.185	+/-	0.0858
**Ankle Positive Work Non Paretic (J kg^{-1})**	0.210	+/-	0.066	0.208	+/-	0.064	0.209	+/-	0.0613	0.206	+/-	0.0485	0.197	+/-	0.0501
**Ankle Positive Work Paretic (J kg^{-1})**	0.081	+/-	0.045	0.079	+/-	0.041	0.091	+/-	0.0550	0.089	+/-	0.0548	0.090	+/-	0.0585
**EXO Positive Work (J kg^{-1})**	0.000	+/-	0.000	0.002	+/-	0.001	0.023	+/-	0.0180	0.018	+/-	0.0098	0.020	+/-	0.0124
**Ankle Negative Work Non Paretic (J kg^{-1})**	-0.269	+/-	0.094	-0.265	+/-	0.074	-0.282	+/-	0.0966	-0.298	+/-	0.0907	-0.295	+/-	0.1014
**Ankle Negative Work Paretic (J kg^{-1})**	-0.158	+/-	0.048	-0.185	+/-	0.056	-0.162	+/-	0.0558	-0.155	+/-	0.0463	-0.161	+/-	0.0514
**EXO Negative Work (J kg^{-1})**	0.000	+/-	0.000	-0.002	+/-	0.002	-0.012	+/-	0.0094	-0.009	+/-	0.0086	-0.007	+/-	0.0056
**Knee Positive Work Non Paretic (J kg^{-1})**	0.077	+/-	0.048	0.076	+/-	0.050	0.076	+/-	0.0607	0.086	+/-	0.0657	0.096	+/-	0.0671
**Knee Positive Work Paretic (J kg^{-1})**	0.081	+/-	0.054	0.085	+/-	0.068	0.089	+/-	0.0667	0.089	+/-	0.0727	0.088	+/-	0.0706
**Knee Negative Work Non Paretic (J kg^{-1})**	-0.268	+/-	0.119	-0.249	+/-	0.099	-0.235	+/-	0.0930	-0.240	+/-	0.0952	-0.241	+/-	0.0828
**Knee Negative Work Paretic (J kg^{-1})**	-0.129	+/-	0.034	-0.126	+/-	0.030	-0.120	+/-	0.0242	-0.116	+/-	0.0185	-0.118	+/-	0.0261
**Hip Positive Work Non Paretic (J kg^{-1})**	0.363	+/-	0.097	0.368	+/-	0.143	0.379	+/-	0.1393	0.398	+/-	0.1508	0.356	+/-	0.1531
**Hip Positive Work Paretic (J kg^{-1})**	0.169	+/-	0.101	0.178	+/-	0.092	0.180	+/-	0.0803	0.193	+/-	0.0805	0.171	+/-	0.0776
**Hip Negative Work Non Paretic (J kg^{-1})**	-0.029	+/-	0.027	-0.026	+/-	0.017	-0.035	+/-	0.0249	-0.032	+/-	0.0237	-0.037	+/-	0.0261
**Hip Negative Work Paretic (J kg^{-1})**	-0.034	+/-	0.020	-0.034	+/-	0.017	-0.031	+/-	0.0208	-0.030	+/-	0.0260	-0.023	+/-	0.0116
